# Shear-free mixing to achieve accurate temporospatial nanoscale kinetics through scanning-SAXS: ion-induced phase transition of dispersed cellulose nanocrystals†

**DOI:** 10.1039/d0lc01048k

**Published:** 2021-01-25

**Authors:** Tomas Rosén, Ruifu Wang, HongRui He, Chengbo Zhan, Shirish Chodankar, Benjamin S. Hsiao

**Affiliations:** Department of Chemistry, Stony Brook University Stony Brook New York 11794-3400 USA benjamin.hsiao@stonybrook.edu; Department of Fiber and Polymer Technology, KTH Royal Institute of Technology SE-100 44 Stockholm Sweden trosen@kth.se; Wallenberg Wood Science Center, KTH Royal Institute of Technology SE-100 44 Stockholm Sweden; National Synchrotron Light Source II, Brookhaven National Lab Upton NY USA

## Abstract

Time-resolved *in situ* characterization of well-defined mixing processes using small-angle X-ray scattering (SAXS) is usually challenging, especially if the process involves changes of material viscoelasticity. In specific, it can be difficult to create a continuous mixing experiment without shearing the material of interest; a desirable situation since shear flow both affects nanoscale structures and flow stability as well as resulting in unreliable time-resolved data. Here, we demonstrate a flow-focusing mixing device for *in situ* nanostructural characterization using scanning-SAXS. Given the interfacial tension and viscosity ratio between core and sheath fluids, the core material confined by sheath flows is completely detached from the walls and forms a zero-shear plug flow at the channel center, allowing for a trivial conversion of spatial coordinates to mixing times. With this technique, the time-resolved gel formation of dispersed cellulose nanocrystals (CNCs) was studied by mixing with a sodium chloride solution. It is observed how locally ordered regions, so called tactoids, are disrupted when the added monovalent ions affect the electrostatic interactions, which in turn leads to a loss of CNC alignment through enhanced rotary diffusion. The demonstrated flow-focusing scanning-SAXS technique can be used to unveil important kinetics during structural formation of nanocellulosic materials. However, the same technique is also applicable in many soft matter systems to provide new insights into the nanoscale dynamics during mixing.

## Abstract 2

We present a shear-free flow-focusing mixing experiment to study time-resolved reaction kinetics of nanoscale materials through scanning-SAXS.

## Graphical abstract

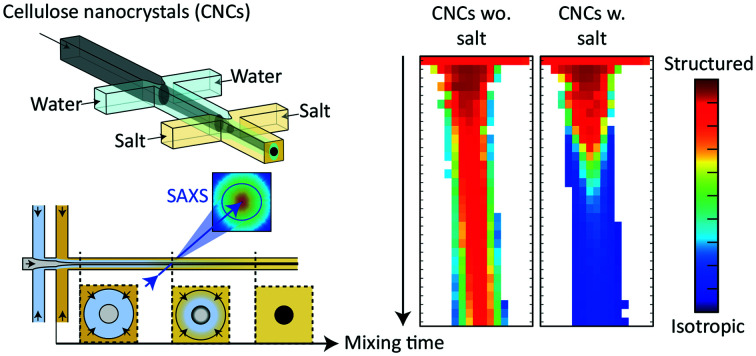

## 1 Introduction

As the performance of new synchrotron X-ray sources has been dramatically enhanced during the past decade, the opportunities of using synchrotron X-rays to study soft materials on a nanometer scale become almost endless, owing to the flexibility to investigate samples under various conditions and the ability to study a large parameter space. Small-angle X-ray scattering (SAXS) is one of the powerful synchrotron techniques to characterize interactions and spatial structures of material on the length scale between 1–100 nm, which has been widely adopted in material, medical, biological and chemical sciences.^1^ In soft matter systems, SAXS can yield structural information regarding the size, shape and ordering of the individual nanoscale entity (*i.e.* the form factor) in dilute systems, including polymers,^2^ proteins,^3^ viruses,^4,5^ nanofibers^6,7^ and other nanoparticles.^8^ In addition, the high brilliance and tunability of synchrotron X-rays allowed the sample environments to be tailored for new *in situ* studies, including *e.g.* magnetic fields,^9,10^ flow deformations^11,12^ and mixing.^13^ The development of micro-focusing X-ray beam capability further enabled the ability of spatial mapping to investigate the structural variations in samples with micrometer-resolution using the scanning-SAXS technique, demonstrated both for solid samples in 2D^14–16^ and 3D,^17,18^ as well as in flows.^11,19^

The combination of SAXS with various mixing experiments has already been seen as a powerful tool to obtain time-resolved transitions including kinetics of RNA or protein folding.^20–22^ Resolving the structural kinetics in traditional mixing experiments using stopped-flow techniques^23^ is inherently problematic because the influence of the radiation itself needs to be corrected (the system often requires to be irradiated for a sufficient period of time to ensure the desired signal-to-noise ratio). The combined SAXS/stopped-flow mixing technique is thus usually more suitable for studies with transitions on time scales of several minutes or more, and for systems where irradiation-induced sample damage is not prominent. To study the short-time kinetics, various microfluidic turbulent and laminar mixing devices, compatible for SAXS experiments, have been demonstrated. In these devices, the sample is continuously mixed in a steady state and the irradiated location can correspond to different time instances of the mixing process.^13^

However, the conversion from spatial coordinates to time instances in continuous flow mixers requires a flow situation with minimal shear to avoid the smearing of the probed kinetic times, which can be problematic in standard laminar mixers with two channels containing the mixing reagents.^24–27^ Another challenge in the continuous flow mixing experiment is if the system becomes viscoelastic, *e.g.* a transition from a viscous dispersion to an elastic gel, where the flow properties may greatly affect the mixing process. This is because the resulting gel can accumulate on the channel walls and the presence of shear can further decrease the flow stability at the mixing interface.

The flow-focusing mixing, using a core flow of the main substance confined by two sheath flows of the reagent solution, has been demonstrated as a way to reduce the velocity gradients inside the main substance.^20–22,28–33^ In order to avoid pre-mixing during focusing, Park *et al.*^34^ suggested a double-focusing setup, with an intermediate buffer sheath flow to separate the main substance from the reagent solution. Most previous studies with flow-focusing mixing still have a sheared parabolic velocity profile since the core fluid is not detached from the walls in the viewing direction. This results in difficulties in obtaining accurate mixing times at certain spatial locations, since the residence time of the material will vary depending on the streamline distance from the wall, and the information in the beam will be an average over all streamlines. Additionally, the shear flow can also influence the structures during mixing as well as affect the flow stability, especially if the mixing also significantly affects the rheological properties of the material. Consequently, the ideal mixing experiment is the one where shear can be avoided altogether.

Fortunately, given the right range of the effective interfacial tension and viscosity ratio between core and sheath, the core fluid can be detached from the walls and flowing in the center of the channel.^35,36^ Once achieved, the system can provide an ideal continuous mixing situation with practically no shear and constant velocity of the main substance, and where all streamlines in the beam have the same residence time in the channel. This in turn allows a trivial conversion from spatial positions to time instances can be obtained for the study of time-resolved mixing kinetics.

An example, where flow-focusing with a detached core is achievable is the hydrodynamic focusing and spinning of filaments from nanocellulose.^31,37–39^ As an example, by applying the double flow-focusing technique, Mittal *et al.*^37^ demonstrated how the first focusing with the buffer caused an alignment of cellulose nanofibers (CNFs), and a second focusing with a gelation agent could make the CNFs aggregate in an aligned state. Drying this gel thread resulted in filaments exceeding mechanical properties of any known natural material. Nechyporchuk *et al.*^38^ also demonstrated that the same double-focusing technique could also be applied to systems with cellulose nanocrystals (CNCs).

When flow-focusing is applied to spin filaments, the detachment of the core flow and the zero-shear environment is crucial as shear will disrupt the formation. However, as explained previously, this also results in the optimal experiment to study time-resolved kinetics during mixing.

In this work, we have therefore explored the idea to use the same flow-focusing method by Mittal *et al.*^37^ as a generalized mixing experiment to study the time-resolved gel transition of CNC dispersions, triggered by the shielding effect of counter monovalent ions (Na^+^) using the scanning-SAXS technique. Even though there have been studies to describe the nanosized structures of CNFs and CNCs at various electrostatic conditions using SAXS,^40–43^ resolving the kinetics of these transitions in real time provides a deeper insight into the nanoscale interactions leading to optimized material processes.

Although the work is primarily focused on this nanocellulosic system, this demonstration can be used to provide a roadmap for similar experiments of other charged anisotropic colloidal materials.

## 2 The time-resolved mixing experiment

**Fig. 1:**
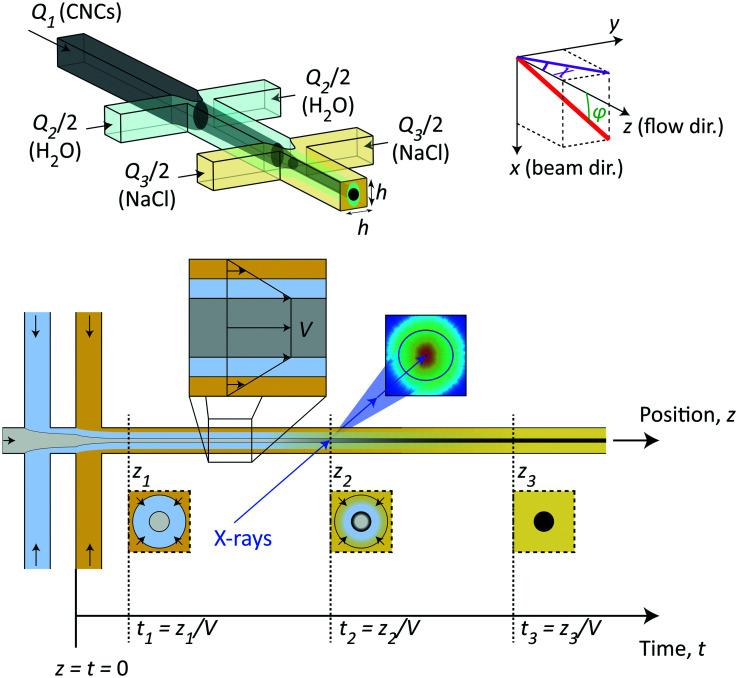
Illustration of the time-resolved mixing experiment used in the study. Dispersed CNCs are flowing in a square channel (side *h* = 1 mm) with flow rate *Q*_1_, which is firstly focused by sheath flows of DI water, each with flow rate *Q*_2_/2 and secondly focused with sheath flows of salt (NaCl) solution, each with flow rate *Q*_3_/2. The addition of salt ions eventually leads to a liquid–gel transition of the CNC dispersions. The system coordinate is defined using *x* and *z* representing the beam and flow directions, respectively. The 3D orientation of a fiber w.r.t. the *z*-axis is given by *ϕ* and the 2D orientation in the viewing (*yz*) plane is given by *χ*. The effective surface tension and higher viscosity of the CNC dispersions yield a plug flow with constant velocity *V* in the core allowing a trivial conversion from spatial coordinates *z* to time instances *t* = *z*/*V* of the gelation process. The time-resolved structural transition is studied using SAXS by focusing the beam at different downstream positions of the flow.

The method in this study relies on using the flow-focusing system demonstrated previously to align and assemble nanocellulosic materials through an ion-induced gelation process,^37,44,45^ as illustrated in Fig. 1. In brief, a flow of dispersed negatively charged CNCs (flow rate *Q*_1_) is firstly focused by sheath flows of water in two perpendicular channels (each with flow rate *Q*_2_/2) and subsequently focused by sheath flows of a gelation agent (each with flow rate *Q*_3_/2), which in this case is a salt solution having monovalent counter ions of Na^+^. All channels have a quadratic cross section with side *h* = 1 mm. In terms of aligning the system, the flow-focusing method has a huge advantage compared to regular wet-spinning techniques as the dispersion in the flow-focusing method is only subject to an extensional flow with very little shear as it is detached from the walls when focused.^39^ While extensional flow causes a steady-state alignment of elongated particles, shear flow can make particles rotate, entangle and aggregate even though the time-averaged state is also partly aligned.^39^ The idea of the double-focusing system is that the dispersion will never be in direct or simultaneous contact with the walls and gelation agent, which can cause gel accumulation and eventually blockage in the channel. The dispersion is aligned from extensional flow in the focusing regions and as the counter ions diffuse through the water layer and eventually reaching the core, the CNC dispersions transition into an arrested (gel) state.

In this study, we used this continuous flow-focusing mixing device to investigate the time-resolved ion-induced gelation behavior in CNC dispersions by *in situ* scanning-SAXS. As a gelation agent, we chose a NaCl solution with concentration of 200 mM. The fact that the core of CNC dispersion was flowing with constant velocity *V* with minimal shear allowed the spatial downstream coordinates *z* to be easily converted to time-instances *t* = *z*/*V* of the gelation process. Focusing the X-ray beam at different spatial locations in the flow, the SAXS pattern have revealed the structure of CNCs at that time instance. Furthermore, by scanning the beam through the core flow, we have been able to determine the spatial distribution of structures inside the core, which provides a deeper insight into the ion diffusion inside the CNC dispersion during the phase transition.

### 2.1 Choosing flow rates

**Fig. 2:**
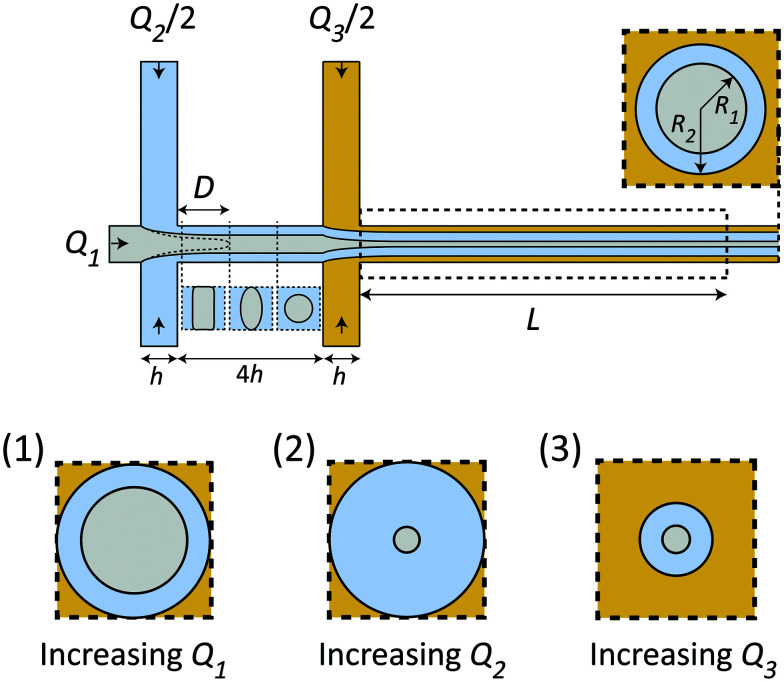
Schematic illustration showing how flow rates influence the flow and time scales. Changing the ratios between the flow rates *Q*_1_, *Q*_2_ and *Q*_3_ changes the radii *R*_1_ (CNCs) and *R*_2_ (water). Changing the ratio *Q*_2_/*Q*_1_ affects the wetting length *D*, where *D* < 4*h* is required for the flow to be detached from the walls before the second focusing. The visible region of the flow is given by *L* = 30 mm. Effect of changing flow rates: (1) increasing only *Q*_1_ leads to a thicker core; (2) increasing *Q*_2_ leads to a thicker water layer and thinner core; (3) increasing *Q*_3_ leads to thinner core and water layer.

Given the certain CNC dispersion and gelation agent, the only control of the system is through the flow rates *Q*_1_, *Q*_2_ and *Q*_3_, whereby setting these rates correctly is essential for a successful experiment as shown in Fig. 2. Increasing the total flow rate at the outlet *Q*_1_ + *Q*_2_ + *Q*_3_ will mainly influence the core velocity *V* and thus increase the time resolution of the experiment, but will also limit the range as the visible region is fixed to *L* = 30 mm (maximum time scale given by *t*_conv._ = *L*/*V*). Ideally, the system should run at very low flow rates to ensure a large *t*_conv._. However, practically, it is found very difficult to control the flow at very low flow rates as the inertia of the flow can aid in stabilizing the flow at higher rates. Changing the ratio between the flow rates will affect the core flow radius *R*_1_ and the radius of the diffusion layer of water *R*_2_, which in turn influences the ion diffusion time scale *t*_ion_, *i.e.* the time for ions to diffuse into the core flow. Simultaneously, if *R*_1_ is too small, the effective concentration (which scales with a factor 2*R*_1_/*h*) inside the X-ray beam will be too low, and the signal-to-noise ratio also becomes very low as the scattering contrast from the dilute nanocellulose dispersion is very close to that from water. Generally, increasing *Q*_1_ (*Q*_2_ & *Q*_3_ fixed) leads to both larger *R*_1_ and *R*_2_ (see inset 1 in Fig. 2), increasing *Q*_2_ leads to a smaller *R*_1_ but larger *R*_2_ (see inset 2 in Fig. 2) and increasing *Q*_3_ leads to both smaller *R*_1_ and *R*_2_ (see inset 3 in Fig. 2).

The flow rate ratio in the first focusing *Q*_2_/*Q*_1_ needs to be initially set to ensure that the wetting length *D* (see Fig. 2) is shorter than the distance between the two focusing channels 4*h*. This is a requirement for the system to run continuously and avoid unwanted gelation at the walls. It was found experimentally that a ratio of *Q*_2_/*Q*_1_ = 2 seemed to fulfill this requirement for a wide range of flow rates. Similarly, the flow rate of the second focusing (*Q*_3_) needs to be set in order to maximize the scattering contrast (*R*_1_) and *t*_conv._, but still allowing the gelation agent to cover the walls and yield an even concentration gradient in all directions around the core. With all these considerations in mind, it was found that the same flow rate in all five inlets was a good compromise. Throughout this work, we thus used the flow rate *Q* to describe the flow rates in all channels, *i.e. Q*_1_ = *Q*_2_/2 = *Q*_3_/2 = *Q*. The slowest flowrates that we could practically handle in this work was *Q* = 1 mL h^−1^, and therefore this was chosen in the experiment in order to maximize the mixing time.

### 2.2 Predicting the Na^+^ concentration profile

In order to estimate the mean concentration 〈*c*〉 of Na^+^-ions in the core CNC dispersion at different positions and flow rates *Q*, a radial ion diffusion simulation was performed using the following assumptions:

1. The flow downstream of the focusing sections is equivalent to a cylindrical system with outer radius 
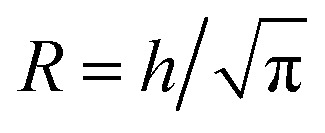
, resulting in the same average velocity for a given flow rate *Q*.

2. The flow is a plug flow with constant velocity *V* at *r* ≤ *R*_1_ and a linear shear in the annulus *R*_1_ ≤ *r* ≤ *R* (*r* being the radial coordinate from the centerline).

3. The diffusivity of Na^+^-ions is the same in water as in the CNC dispersion and given by *D*_ion_(Na^+^) = 1.33 × 10^−9^ m^2^ s^−1^.

4. Initial Na^+^ concentration is *c* = 200 mM at *R*_2_ ≤ *r* ≤ *R* and zero everywhere else.

**Fig. 3:**
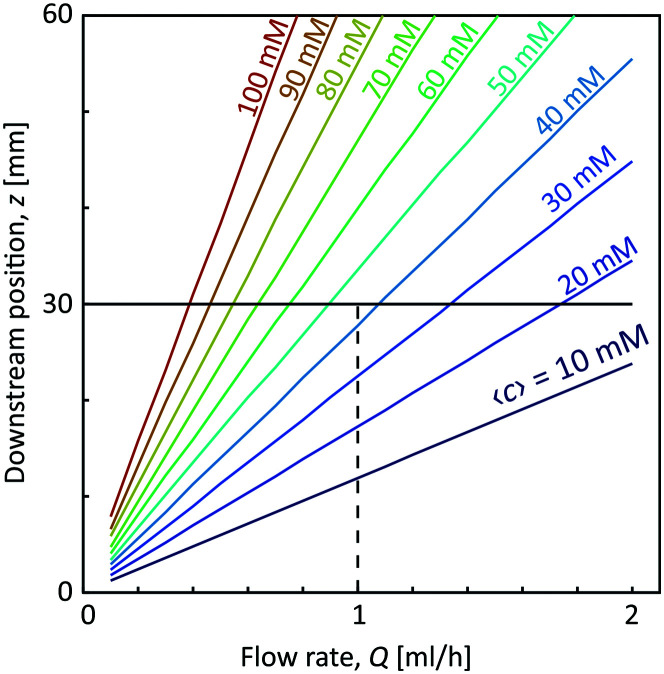
Predicted mean concentration 〈*c*〉 of Na^+^-ions in the core flow at different flow rates *Q* = *Q*_1_ = *Q*_2_/2 = *Q*_3_/2 and downstream positions *z*. The dashed line shows the flow rate used in this work (*Q* = 1 mL h^−1^).

With the first two assumptions, the radii *R*_1_ and *R*_2_ as well as the core velocity *V* can be calculated by knowing the flow rates. Since the radii only depend on the ratio between the flow rates in the different channels, they remain the same regardless of *Q* with values *R*_1_/*h* = 0.17 and *R*_2_/*h* = 0.32. Using the radial diffusion equation, we can determine the mean concentration 〈*c*〉 in the core (*r* < *R*_1_) at the given time instance. By further knowing the velocity *V*, the time-dependent results can be converted to spatial positions through *z* = *Vt*. Fig. 3 shows the results from this analysis. At a flow rate of *Q* = 1 mL h^−1^ for CNC, we predict a core concentration of up to 〈*c*〉 = 40 mM within the visible region (*L* = 30 mm). Further details of the simulation procedure are provided in the Methods section.

## 3 Results and discussion

### 3.1 Sample characterization

**Fig. 4:**
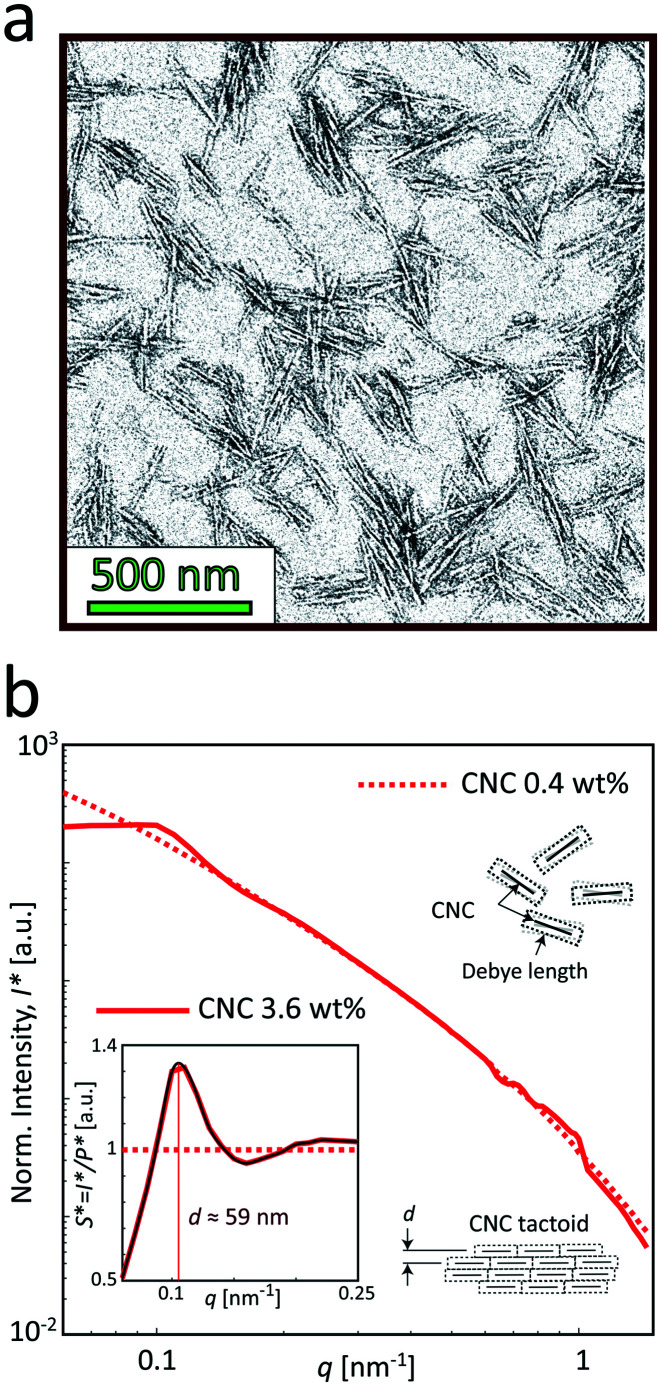
Characterization of the CNC dispersions used in the study (identical dispersions were used by Rosén *et al.*^11^). (a) A typical TEM image of CNCs (re-printed with permission). (b) The SAXS intensity profiles I* normalized with invariant Q* (see Materials and methods section) of the CNC dispersions at 0.4 and 3.6 wt%. Inset shows the structure factor S* = I*/P*, where the main peak corresponds to the average distance between nanocrystals in the tactoidal state. The apparent structure at 0.5 < *q* < 1 nm^−1^ is found to be remaining detector artifacts after azimuthal averaging.

The samples used in this study was commercial CNCs (produced through acid hydrolysis) made from bleached wood pulp provided by the Process Development Center in University of Maine. The same material was used and characterized in the work by Rosén *et al.*^11^ The TEM image of the material in Fig. 4a shows the CNCs having lengths approximately in the range of 150–300 nm and widths at 10–30 nm as previously determined.^11^ The sample in the study was prepared at 3.6 wt%. At this concentration, the dispersion is in a semi-dilute isotropic tactoidal state due to the electrostatic interactions of the nanocrystals.^9,11,46–48^ More details about the samples is provided in the Materials and methods section.

Fig. 4b shows the SAXS profiles from isotropically distributed CNC dispersions. At the low concentration (0.4 wt%), the profile is dominated by the scattering from the cross-sections of individual nanoparticles, and therefore can be used to determine the form factor *P*(*q*) of the individual CNC particle (dashed curve). At the high concentration (3.6 wt%), where the dispersion is in the isotropic tactoidal state (solid curve), the average distance between particles is similar to the Debye length (characterizing how far from the charged particle where the electrostatic forces persist), giving rise to an organized structure due to electrostatic interactions. The transition to the tactoidal state becomes clearly visible in the SAXS data of the material in Fig. 4b, showing the normalized scattering intensity I*(*q*) (normalized with invariant Q*; see Materials and methods section for details). Dividing I*(*q*) with the normalized form factor P*(*q*), we get the structure factor S*(*q*) shown in the inset figure. Plotting the data in this way provides us an indication of how the spatial configuration of nanocrystals differ from a disordered (dilute) system and can consequently be used to study the structural changes during gelation. The prominent peak at *q* ≈ 0.11 nm^−1^ is indicating the length scale of the structures *d* = 2π/*q* ≈ 59 nm, which is corresponding to the average distance between CNCs in the tactoids.^42,43,46^

### 3.2 Flow cell

The flow cell consists of a 1 mm aluminum channel plate with the double-focusing geometry placed between two films made of cyclic olefin copolymer (COC) serving as window material. The cell is mounted together using 10 mm aluminum plates with slits to allow visual access (see Fig. S1 in the ESI†). The main flow direction *z* is aligned with the direction of gravity and the flow is led directly into a container with water. For more details on the experimental flow cell and setup, see the Materials and methods section and Fig. S2 in the ESI.†

Prior to the *in situ* SAXS experiments, the flow was observed using polarized optical microscopy (POM) and placing the flow cell between two cross-polarized linear polarization filters (see Fig. S3 in the ESI† and ESI† Video). In this way, we could determine that we indeed have the formation of a gel in the system and, owing to small impurities in the flow, confirm that the flow is indeed a plug flow (all impurities moving with a constant velocity) as well as to experimentally determine the core velocity *V* and the projected radius *R*_1,*y*_. By knowing the core flow rate *Q*_1_, we can calculate the core radius in the viewing direction *R*_1,*x*_ through *Q*_1_ = π*R*_1,*x*_*R*_1,*y*_*V*. The comparison between the experimental values and the values from the prior analysis is shown in Table 1.

**Table 1:** Comparison of core flow radii *R*_1,*x*_ and *R*_1,*y*_ and velocity *V* between predictions and the experimentally determined values from the POM experiments (see ESI† for details)

	*R*_1,*x*_/*h*	*R*_1,*y*_/*h*	*V* [mm s^−1^]
CNC *Q* = 1 mL h^−1^ (predicted)	0.17	0.17	2.98
CNC *Q* = 1 mL h^−1^ (experimental)	0.15	0.17	3.47

**Fig. 5:**
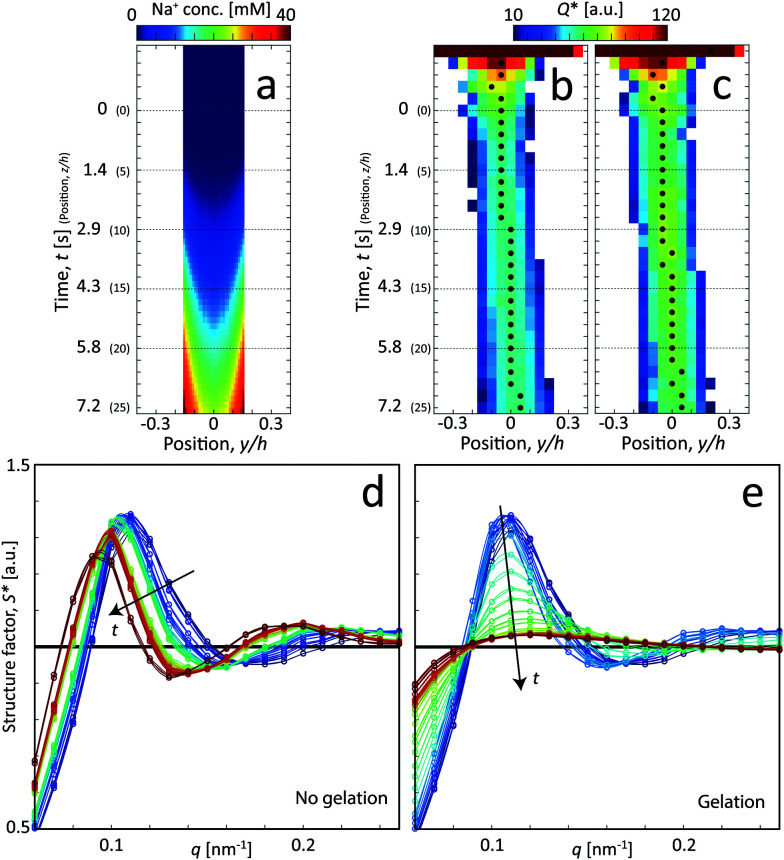
Analysis of the scanning-SAXS of CNCs. (a) Mean Na^+^ concentration in the core from the ion diffusion simulation averaged in the beam (*x*) direction. (b) and (c) Invariant Q* depending on the position of *z* and *y*, where the downstream position *z* is converted to time using *t* = *z*/*V* for flow: (b) with no gelation, and (c) with gelation. The markers indicate the positions of maximum Q* used as the centerplane data. (d) and (e) Structure factor S* along the centerplane, *i.e.* depending on time *t* (indicated by arrows) for flow with (d) with no gelation, and (e) with gelation.

Interestingly, the estimations and the true values are quite similar, where the actual core velocity is slightly underestimated. The experimental values reveal that the core flow is fairly circular (*R*_1,*x*_ ≈ *R*_1,*y*_), but the discrepancies indicate that the velocity profile probably is a bit different in the square channel compared to the simplified cylindrical geometry used in the predicted system. Nevertheless, the simulation procedure provided in section 2.2 can be concluded to be a very good method to provide estimations of the time resolution of the experiment. Using these experimentally determined values of *R*_1_ and *V*, the Na^+^ concentration profile was refined accordingly (see Fig. S4 in ESI†). In order to compare the simulations with the scanning-SAXS results, the Na^+^ concentration in the core (*r* < *R*_1_) was averaged in the beam (*x*) direction in Fig. 5a.

### 3.3 *In situ* SAXS experiments

The flow cell was set up at the LiX beamline (16-ID) at the National Synchrotron Light Source II (NSLS-II) at Brookhaven National Laboratory (BNL), USA. The system was mounted on a traversing stage, allowing for *in situ* scanning-SAXS measurements where the beam is focused at different locations in the channel. An illustration of how these experiments were performed is provided in Fig. S5 in the ESI.†

Complementary to these scanning-SAXS experiments, we also analyzed pre-mixed samples of CNCs and NaCl to compare structures in systems with known charge ratios (see Table 2).

**Table 2:** List of pre-mixed samples used to compare with the *in situ* scanning-SAXS results

#	Sample	Surface charge [mmol mL^−1^]	Na^+^ added [mmol mL^−1^]	Charge ratio
1	CNC 3.6 wt%	0.011	0.006	0.5
2	CNC 3.6 wt%	0.011	0.012	1.1
3	CNC 3.6 wt%	0.011	0.024	2.2

More details about the measurements are provided in the Methods section and in the ESI.†

#### 3.3.1 Scanning-SAXS of CNCs during flow-focusing mixing

For the scanning-SAXS experiments, the invariant Q* is initially calculated for every SAXS image at each position to provide a scanning image of the flow, as illustrated for CNCs without and with gelation in Fig. 5b and c, respectively. As the invariant is a measure of the amount of CNC material in the X-ray beam, we can estimate the centerplane *y*-location at each downstream position *z* as the position with highest invariant (noted with black symbols in Fig. 5b and c). In these figures, it is also observed that the invariant is higher for gelled CNCs, which is likely an indication of a higher local concentration of CNCs in the gel.

For the centerplane data, the structure factor S* is extracted in order to track what happens to the CNC tactoidal structure without and with gelation in Fig. 5d and e, respectively.

Without gelation, the structure remains more or less intact over time, but where the main structure peak moves towards lower *q* as the local concentration of CNCs slowly decreases as they diffuse out from the core. The lower local concentration also leads to a larger distance between CNC particles in the tactoids. With gelation through addition of NaCl, the CNC nanostructure is clearly lost and the scattering profile approaches the form factor (S* = constant). As the Na^+^ ions diffuse into the core, they screen the electrostatic surface charges that give rise to the formation of locally ordered tactoids and the CNCs become more disordered as a consequence.

**Fig. 6:**
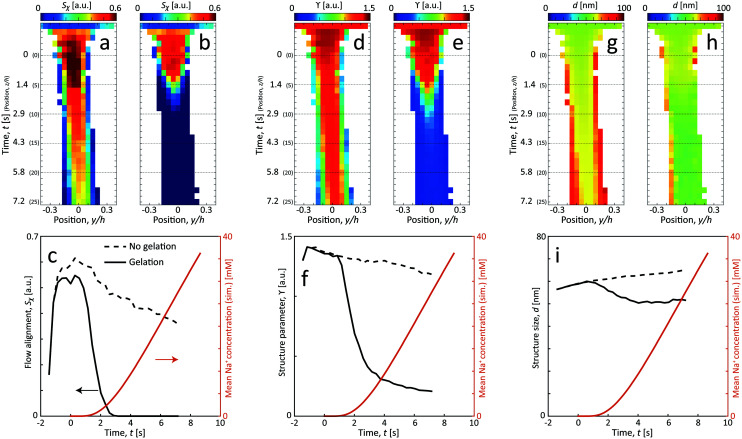
Results of the scanning-SAXS of CNCs. (a) and (b) CNC alignment *Sχ*, (d) and (e) CNC structure parameter *ϒ* and (g) and (h) CNC structure size *d* for flow (a), (d) and (g) with no gelation, and (b), (e) and (h) with gelation. (c), (f) and (i) The same parameters evaluated along the centerplane and compared with the simulated mean Na^+^ concentration 〈*c*〉 (see ESI† for details of simulation).

In Fig. 6, the CNC structure is quantified using three parameters (detailed definition in section 5):

1. The order parameter *S*_*χ*_, quantifying the average CNC alignment in the flow direction.

2. The structure parameter *ϒ*, quantifying the deviation from the form factor and thus provides a measure of how structured the material becomes.

3. The structure size *d*, quantifying the length scale of the main structure peak.

As noted in the work by Rosén *et al.*,^11^ CNCs in the tactoidal state can be easily aligned even at very low flow rates, which is observed in Fig. 6a–c with the order parameter reaching as high as *S*_*χ*_ = 0.6 in the second focusing region (*z* ≈ 0). Rosén *et al.*^11^ noted that while dilute CNCs are highly dominated by the Brownian rotary diffusion and are difficult to align, the formation of tactoids lead to the suppression of thermal motion making them easier to align. It is very interesting to observe that as the Debye length of the CNCs decreases with the addition of NaCl, the tactoids are destroyed and the particles become dominated by the Brownian rotary diffusion. As a consequence, any flow alignment rapidly vanishes and is gone after *t* = 2–3 s, *i.e.* at *z*/*h* = 5–10 (the same loss of alignment can also be observed in POM experiments, see Fig. S3 in ESI† and ESI† Video).

Fig. 6d–i show how the structure changes in the flow. Without gelation, the structure parameter *ϒ* decreases slowly and steadily while the average distance *d* between CNCs in the tactoids increases. From the scanning images, it is clear that the effect is even quicker at the interface between CNCs and water, as the CNC dispersion gets diluted, the nanostructure changes accordingly due to a lower local concentration. Assuming that the interparticle distance increases according to the volumetric change, the following scaling should hold: *d* ∝ *c*^1/3^_CNC_. Knowing that initially the concentration is *c*_CNC,0_ = 3.6 wt% and *d*_0_ = 59 nm, the local concentration can be estimated through the measurement of *d*. As an example, the concentration after *t* = 7.2 s at the centerplane can be estimated as *c*_CNC_ = 3.6·(59/65)^3^ = 2.7 wt%. Furthermore, using the steady increase of *d* in Fig. 6i with respect to time, the translational diffusion coefficient of CNCs can be estimated: *D*_CNC_ = 1.25 × 10^−10^ m^2^ s^−1^ (details provided in section 5).

With gelation, as noted before, the structure is lost throughout the core flow cross-section. During this transition from aligned tactoids to an isotropic gel, the structure peak is shifted slightly towards smaller *d*, indicating how CNC particles approach each other to form a gel with a higher local concentration.

#### 3.3.2 Comparison with pre-mixed samples

**Fig. 7:**
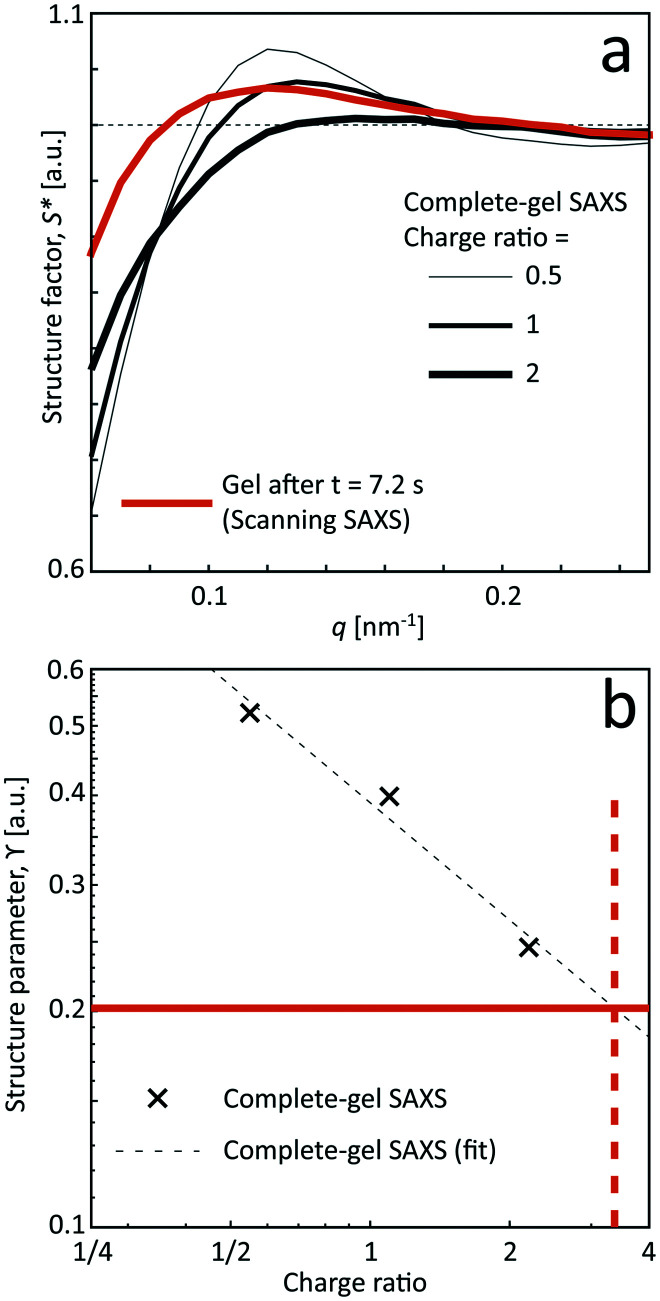
Results from the SAXS of the pre-mixed gel samples. (a) Structure factor S* and (b) structure parameter *ϒ* as a function of the charge ratio of the added Na^+^ ions compared to the surface OSO_3_^−^ groups. The orange curve shows the data of S* and *ϒ* at the most downstream location (*z*/*h* = 25, *t* = 7.2 s) in the scanning-SAXS experiments. By extrapolating the data of the structure parameter *ϒ* against charge ratio, the approximate charge ratio is close to 3 at the most downstream location in the flow, corresponding to a Na^+^ concentration of *c* = 33 mM.

The previous section reveals the kinetics of the CNC gel formation as the NaCl solution is added to the system. As a comparison, gels were also prepared by mixing the dispersion with different amounts of NaCl as described in Table 2 and leaving it for more than 12 hours prior to the measurements. The structure factor S* of the pre-mixed samples are provided in Fig. 7a and compared with the results from the scanning-SAXS experiments at the most downstream location (*z*/*h* = 25, *t* = 7.2 s).

Even though seemingly forming a gel at the charge ratios of 0.5, 1 and 2, the corresponding structures are a bit different compared to the flowing system as evident from the structure factor results. Although the pre-mixed samples also show that tactoids are vanishing with increasing salt concentration, the structure is clearly more disordered in the flowing system in the scanning-SAXS experiment. Furthermore, the pre-mixed gel seems to become more compact, as the structure peak is shifted to a higher *q*-value. However, the deviation from S* = 1 is still quite large at low *q*, indicating the existence of larger scale structures, which cannot be observed in the scanning-SAXS experiment.

In Fig. 7b, the structure parameter *ϒ* is plotted against the charge ratio, which seems to be fairly well described with an exponential function in the range of the study. Assuming that *ϒ* is a good indicator of the local charge ratio, the Na^+^ concentration can be estimated at different positions in the scanning-SAXS experiment. At the most downstream location in the scanning-SAXS experiment (*z*/*h* = 25, *t* = 7.2 s), where *ϒ* ≈ 0.2, this would roughly correspond to a charge ratio of 3 or *c* ≈ 33 mM. This is quite close to the simulated mean Na^+^ concentration in the core 〈*c*〉 (see orange curve in Fig. 6), which is remarkable given the simplicity of the model. Although further analysis is needed to verify these results, we hypothesize that the structure factor in the flow can be used as an indicator of the local salt concentration, and thus can be used to provide better models of the ion diffusion in the flow-focusing mixing process.

### 3.4 A proposed transition scenario

Using the results from the previous sections, a likely scenario for the nanostructural changes of the CNC dispersion during Na^+^ induced gelation will be provided here.

**Fig. 8:**
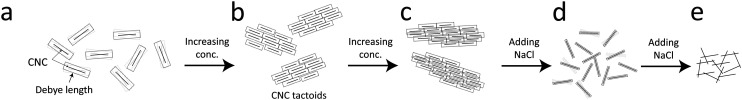
Illustration of the hypothesized scenario during CNC gel formation. (a) In the dilute state, CNCs are moving independently as the Debye length is much smaller than distances between the particles. (b) With increasing concentration, CNCs form locally ordered larger objects called tactoids. (c) With further increasing concentration, the tactoids become more closely packed. (d) With salt addition, the Debye length decreases and electrostatic interaction forming tactoids disappears; CNCs start moving independently. (e) Further addition of salt, van der Waals forces become dominant, causing CNCs to aggregate and form a gel.

A proposed scenario is illustrated in Fig. 8. The interactions between CNCs are dominated by electrostatic forces with a distance that can be characterized by the Debye length. In the dilute state in Fig. 8a, the average distance between the particles is much greater than the Debye length and they tend to move independently. The charged CNCs behave approximately as small Brownian ellipsoids,^11^ which are difficult to align as the hydrodynamic forces must overcome the Brownian rotary diffusion. With the increase in concentration, CNCs can form tactoids of locally ordered particles^11^ (Fig. 8b), although the Brownian motion still tends to keep the overall system isotropic. Owing to the much larger scale of the tactoids, these objects are much less Brownian than individual CNCs, where the tactoids can be easily aligned even with small deformation rates. Increasing the concentration further, the tactoids become more densely packed and can also grow in size (Fig. 8c). At much higher concentrations than used here, the system will become cholesteric, where the entire system is anisotropic.^47^

In the present work, without gelation, the system is slowly diluted due to the translational diffusion of CNCs into the surrounding water, leading to a slow transition from the closely packed tactoids in Fig. 8c to less packed tactoids in Fig. 8b. With the addition of NaCl to the system, the Na^+^ ions can diffuse towards the negatively charged CNCs and screen its surface charges, effectively decreasing the Debye length (Fig. 8d). With the decrease in Debye length, the tactoids gradually become smaller and fewer with increasing salt concentration. Eventually, the tactoids disappear resulting in CNCs moving more individually. In the present work, as the hydrodynamic force is so weak, CNCs will quickly approach an isotropic state as tactoids become smaller due to the Brownian rotary diffusion. Further adding salt to the system (Fig. 8e), the electrostatic forces will be negligible and the CNCs aggregate to form a gel due to van der Waals forces.

## 4 Conclusions

The main purpose of this work is to demonstrate how time-resolved nanoscale structural transitions can be characterized by combining shear-free flow-focusing mixing and scanning-SAXS. Owing to the large viscosity difference between the core and the sheath fluids, a plug flow with a uniform velocity profile is formed in the core, which allows for mixing only through diffusion without shear. The plug flow condition enables the trivial conversion of downstream spatial positions into time instances of the mixing process. The plug flow profile also makes this kind of setup ideal to study phenomena where the mixing also leads to rheological thickening of the core fluid.

To demonstrate this technique, we investigated the transition of dispersed cellulose nanocrystals (CNCs) into a hydrogel with the addition of sodium chloride (NaCl) solution. To attribute the effect of the salt concentration, the same flow was also set up without salt solution as a reference. The nanoscale structure was mainly characterized by extracting the structure factor and orientation distribution from the SAXS images. To estimate the average Na^+^ concentration at each position in the flow, a simplified ion diffusion simulation was performed using a radial diffusion equation. Furthermore, the results using scanning-SAXS were compared with the measurements of pre-mixed gels with known amounts of salt solution.

At the chosen concentration of 3.6 wt%, CNCs are arranging in locally ordered tactoids, randomly distributed in the dispersion. The tactoids are easy to align at low flow rates with the sheath flow of pure water. However, these tactoids are gradually disappearing due to the translational diffusion of CNCs into the sheath. Without gelation, this experiment revealed the rate of this diffusion. With addition of salt solution in the sheath flow, the tactoidal state was disappearing and CNCs rapidly reach isotropy due to the increase in Brownian rotary diffusion. Eventually, as the Na^+^ ions are screening the charges on the CNC surface, nanocrystals can aggregate and form a gel. However, since the tactoid destruction is much quicker than the gel formation, an isotropic CNC gel will be formed even though we reach very high degree of alignment before mixing takes place. Comparing to the pre-mixed gels, we find that the tactoidal structure of CNCs seems to be mainly dependent on the local NaCl concentration, providing a possibility to estimate the local salt concentration through the structure factor, which is close to the simulated value.

With this technique, we foresee that similar studies of high aspect ratio cellulose nanofibrils (CNFs) could provide a greater insight into the hydrogels used for strong and stiff cellulose filaments. It would also be interesting to study how the nanostructural transition depends on different gelation agents (*e.g.* multivalent counter ions) and different CNC concentrations.

Nevertheless, as a final remark, we would like to emphasize that this type of experimental setup is by no means restricted to the systems of nanocellulose. We predict that there likely are many other applications in both material science and life science, where shear-free flow-focusing combined with scanning-SAXS can reveal new interesting time-resolved phenomena on a nanometer scale. In some cases, we hypothesize that it could be appropriate to adjust the core flow solvent to achieve the interfacial tension needed to provide a detached core and provide the same shear-free mixing as demonstrated in this work.

## 5 Materials and methods

### 5.1 Sample preparation

An aqueous slurry of cellulose nanocrystals (CNCs) from bleached wood pulp were purchased from the Process Development Center of University of Maine.

The original aqueous 12 wt% CNC slurry was produced through acid hydrolysis, resulting in a charge of 0.3 mmol of sulfate groups (OSO_3_^−^) per gram of solid material. The slurry was diluted to a dispersion of 3.6 wt% (determined through gravimetric analysis), *i.e.* with a charge concentration of 10.8 mM OSO_3_^−^.

Through transmission electron microscopy (TEM) of the material,^11^ determined the lengths of CNCs to be around 150–300 nm. By fitting the dilute SAXS data using a parallelepiped model,^11^ determined the mean cross sections of CNCs to be 30.8 × 3.5 nm^2^.

### 5.2 Flow setup

#### 5.2.1 Flow cell

The flow geometry consisted of five inlets and one outlet according to Fig. 1, where the channels had a quadratic cross-section of 1 × 1 mm^2^. This geometry was milled through a 1 mm thick aluminum plate, which was sandwiched between two 150 μm thick COC films (Tekni-plex 8007 X-04) and mounted together with two outer aluminum plates (see Fig. S1 in ESI†). A core flow of CNC dispersion with flow rate *Q*_1_ was initially focused by a sheath flow of DI water from the first two perpendicular channels, each with a flow rate of *Q*_2_/2. The gelation agent, an aqueous sodium chloride solution (200 mM NaCl), was injected as a second sheath flow from another two perpendicular channels, each with a flow rate of *Q*_3_/2, located 5 mm (distance between channel centers) downstream from the first sheath. The water flow from the second sheath acted as a diffusion layer, through which the Na^+^ ions diffused through to the core of CNCs and caused a liquid–gel transition. The main flow direction was the same as gravity and the flow eventually went straight into an outlet container with DI-water. Given the fact that the flow-rates is much lower than a gravity driven flow, the pressure inside the flow cell is lower than the atmospheric pressure, which causes a slight inwards bulging of the COC-films. The maximum bulging distance is however estimated to be less than 10 μm and thus negligible compared to the 1 mm channel height. Furthermore, the flow geometry described here is identical to the one used in several other works describing controlled alignment and assembly of dispersed cellulose nanofibrils.^37,44,45^

#### 5.2.2 Fluid distribution

The main flow (with flow rates *Q*_1_, *Q*_2_ and *Q*_3_) was driven by three syringe pumps (NE-4000), where the sheath flow pumps possessed two 1 mL syringes (one for each side channel) and the core flow naturally only had one (1 mL) syringe.

As a compromise to get good scattering contrast and ensure a stable flow (described in the main text), the same flow rate was chosen for all small syringes, *i.e. Q*_1_ = *Q*_2_/2 = *Q*_3_/2 = *Q*, leading to a flow rate of 5*Q* in the outlet channel during the measurement.

### 5.3 Radial ion diffusion simulations and time scale estimation

For the time scale analysis, the channel cross-section was assumed to be circular with the radius 
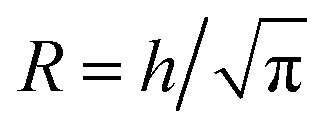
 and thus the same cross-sectional area to match the mean velocity. The flow was assumed to be a plug flow of CNCs at the center with constant velocity *V* in the region *r* ∈ [0, *R*_1_] with *r* being the radial coordinate from the centerline. In the water layer *r* ∈ [*R*_1_, *R*_2_] and the salt layer *r* ∈ [*R*_2_, *R*]. In the annulus with water and salt solution, the velocity was assumed being a linear shear flow. The plug-shear velocity profile in the flow-focusing system has previously been proven a valid assumption in previous work.^49^ By knowing the flow rates *Q*_1_, *Q*_2_ and *Q*_3_, we could calculate the corresponding radii *R*_1_ and *R*_2_ as well as the core flow velocity *V* (see details in ESI†).

To simulate the ion concentration profile in the channel, the diffusion equation for ion concentration *c* is simulated in time according to:1
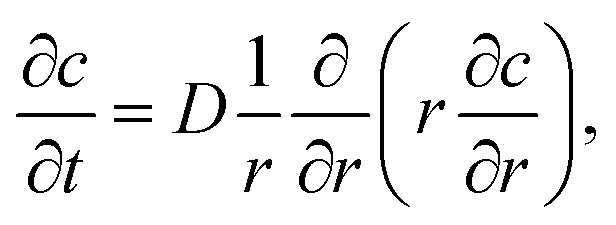
where *D*_ion_ = 1.3 × 10^−9^ m^2^ s^−1^ is the diffusivity Na^+^ in water. Initial conditions are set to *c*(0 ≤ *r* < *R*_2_, *t* = 0) = 0, *c*(*R*_2_ ≤ *r* ≤ *R*, *t* = 0) = *c*_0_ and ion concentration *c*_0_ = 200 mM in outer sheath fluid. The concentration gradient is further set to zero at the boundaries. The mean concentration in the core is calculated through:2
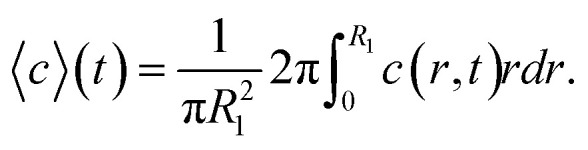
Time instances *t* are then easily converted to spatial coordinates *z* through *z* = *Vt*.

### 5.4 *In situ* SAXS experiments

#### 5.4.1 Synchrotron scattering setup

The *in situ* SAXS experiments were performed at the LiX beamline (16-ID) in the NSLS-II, Brookhaven National Laboratory, USA. The flow cell was mounted on a translation stage in the path of the beam to allow for scanning in both *y*- and *z*-directions (see Fig. S5 in ESI† for photos of the setup).

The flow is running continuously while taking one image with 1 s exposure at various positions in both *y*- and *z*-directions. Each scan of 17 horizontal points at 31 different downstream locations (527 points in total) takes approximately 30 min. In these measurements, the core interface is monitored with an on-axis camera during the entire measurement to ensure the flow stability throughout the measurement.

For the pre-mixed gels, the samples are injected into liquid sample holders, with window materials made from mica, which are scanned at five different positions with 1 s exposure at each position. A second image was scanned directly afterwards at the same position to ensure that there is no beam damage to the sample.

The wavelength was *λ* = 0.9 Å and the sample-detector-distance was 3.6 m. The beam size was approximately 50 × 50 μm^2^. The scattered X-rays were recorded on a Pilatus 1 M detector with pixel size 172 × 172 μm^2^. Simultaneous wide-angle X-ray scattering (WAXS) patterns were also recorded on two Pilatus 300 k detectors (with same pixel size). The SAXS detector was spanning the range of *q* = 0.06–2 nm^−1^, while the WAXS detectors covered *q* = 2–30 nm^−1^, with scattering vector magnitude *q* = (4π/*λ*)sin *θ* (the angle between incident and scattered light is 2*θ*). The background scattering intensity was obtained by flowing only DI water in the channel and subtracted from the experimental data. Prior to this subtraction, the background intensity was scaled with a factor (close to one) corresponding to the ratio of the recorded transmitted beam. Although, the WAXS data was recorded for all the experiments, no conclusive features could be extracted and are therefore not presented here.

#### 5.4.2 Post-processing of scattering data

The SAXS intensity *I*(*q*_*y*_, *q*_*z*_) was converted into polar coordinates *I*(*q*, *χ*) with azimuthal angle *χ* in the detector plane (*χ* = 0 in the *y*-direction). The pixels covered by inter-module gaps and beamstop were corrected using the fact that *I*(*q*_*y*_, *q*_*z*_) = *I*(−*q*_*y*_, −*q*_*z*_). To obtain the orientation distribution function *Ψ*_*χ*_ of the nanofibers from the 2D SAXS data, the same method was applied as done by.^11^ In brief, the isotropic contribution to the scattering *I*_iso_(*q*) was subtracted from the 2D data, with subsequent normalization at each *q* and averaging over a given *q*-range resulting in *Ψ*_*χ*_. The isotropic data *I*_iso_(*q*) is determined by *I*_iso_(*q*) = *I*(*q*_*y*_ = 0, *q*_*z*_ = *q*) from a highly aligned system achieved through single flow focusing at high flow rates. Since the same dispersions were used here, *I*_iso_(*q*) was the same as used by.^11^ Prior to subtraction, the isotropic contribution was scaled using the invariant Q*. Once *Ψ*_*χ*_ is extracted, the alignment is characterized with the order parameter *S*_*χ*_, defined as:3
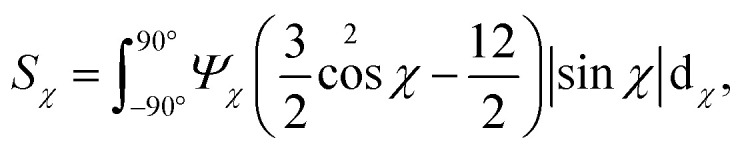
where *Ψ*_*χ*_ is normalized according to:4
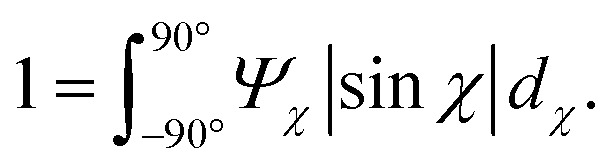
The 1D SAXS data *I*(*q*) is extracted by averaging the 2D data over all angles *χ*. The form factor *P*(*q*) of dilute CNCs is provided from the isotropic (low flow rate) data presented by Rosén *et al.*^11^ from the same CNCs at 0.4 wt%. The intensity data *I*(*q*) from five different measurements is fitted with a spline interpolation in Matlab R2018b to create the curves of *P*(*q*) used to evaluate the structure of the present systems (see Fig. 4b).

The following parameters are derived from the 1D data in this work:

1. Invariant Q*. This is a measure of the total scattering power of the system and is typically proportional to the amount of particles in the beam. The invariant is estimated through:5
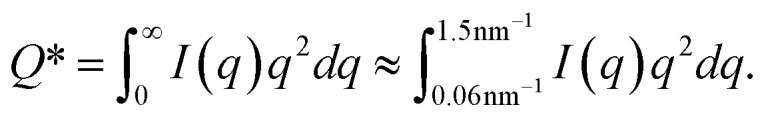
Intensity normalized with the invariant is denoted with an asterisk, i.e. I* = *I*/Q*.

2. Structure parameter *ϒ*. This parameter is used to estimate the deviation from the form factor *P*(*q*) of the system and is calculated through:6
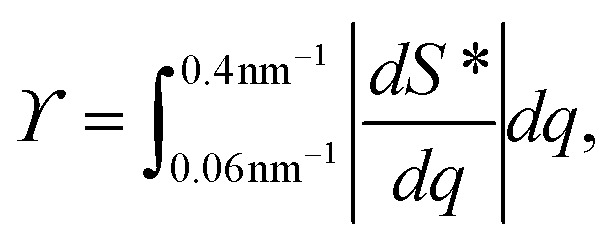
where S* = I*/P*.

3. Structure size *d*. This parameter is used to describe the length scale of the most dominant feature of the structure factor S*. It is evaluated by fitting the structure factor S* to a spline function and finding the maximum S*(*q*_d_) = max(S*) in the range *q* ∈ [0.06, 0.25] nm^−1^. The structure size is defined as *d* = 2π/*q*_d_. In the tactoidal CNC dispersion, this parameter corresponds to the average distance between the nanocrystals.^46^

### 5.5 CNC translational diffusion

The average distances *d* between CNCs in the tactoidal state was determined by the SAXS experiments through the structure factor as described above. This distance should increase with concentration following the relationship of *d* ∝ *c*^−1/3^, *i.e.* the distance increases according to the apparent volumetric change. The local radial concentration *c*(*r*, *t*) of CNCs can be simulated through the radial diffusion equation in eqn (1) with initial conditions *c*(0 ≤ *r* ≤ *qR*_1_, *t* = 0) = *c*_0_ = 3.6 wt% and *c*(*R*_1_ < *r* ≤ *R*, *t* = 0) = 0 and radius *R*_1_ = 0.16 mm determined from experiment (see Table 1). From data in Fig. 6i, it is found that *d*_0_ = 59.0 nm at *t* = 0 and *d*_1_ = 65.14 nm at *t* = 7.2 s. The local distance *d*(*r*, *t*) between CNCs can be calculated through:7
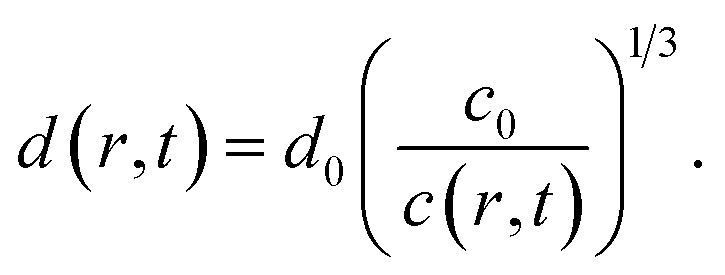
The averaged measured distance in radial direction is weighted by concentration:8
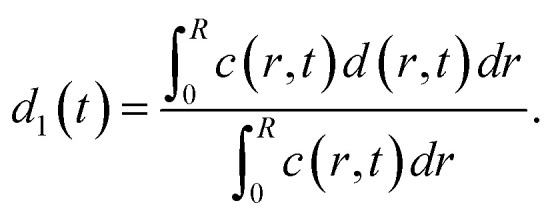
The diffusion coefficient *D* = 1.25 × 10^−10^ m^2^ s^−1^ is then found by matching the time scale of the diffusion equation to match *d*_1_(*t* = 7.2 s) = 65.14 nm.

## Conflicts of interest

There are no conflicts to declare.

## Supplementary Material

LC-021-D0LC01048K-s001

LC-021-D0LC01048K-s002
