# Supplementary material for: Shear-free mixing to achieve accurate temporospatial nanoscale kinetics through scanning-SAXS: ion-induced phase transition of dispersed cellulose nanocrystals
Source: Lab Chip. 2021 Jan 25;21(6):1084–95. doi: 10.1039/d0lc01048k (PMC8323814; doi:10.1039/d0lc01048k)
Supplement: LC-021-D0LC01048K-s002 [file LC-021-D0LC01048K-s002.pdf]

Electronic Supplementary Material (ESI) for Lab on a Chip.
This journal is © The Royal Society of Chemistry 2021

Shear-free mixing to achieve accurate temporospatial nanoscale kinetics through scanning-SAXS: Ion-induced phase transition of dispersed cellulose nanocrystals

Supporting Information

Tomas Rosén,[*,†,‡,¶] Ruifu Wang,[†] HongRui He,[†] Chengbo Zhan,[†] Shirish Chodankar,[§] and Benjamin S. Hsiao[*,†]

†*Department of Chemistry, Stony Brook University, Stony Brook, New York 11794-3400, United States*

‡*Department of Fiber and Polymer Technology, KTH Royal Institute of Technology, SE-100 44 Stockholm, Sweden*

¶*Wallenberg Wood Science Center, KTH Royal Institute of Technology, SE-100 44 Stockholm, Sweden*

§*National Synchrotron Light Source II, Brookhaven National Lab, Upton, NY, USA*

E-mail: trosen@kth.se; benjamin.hsiao@stonybrook.edu

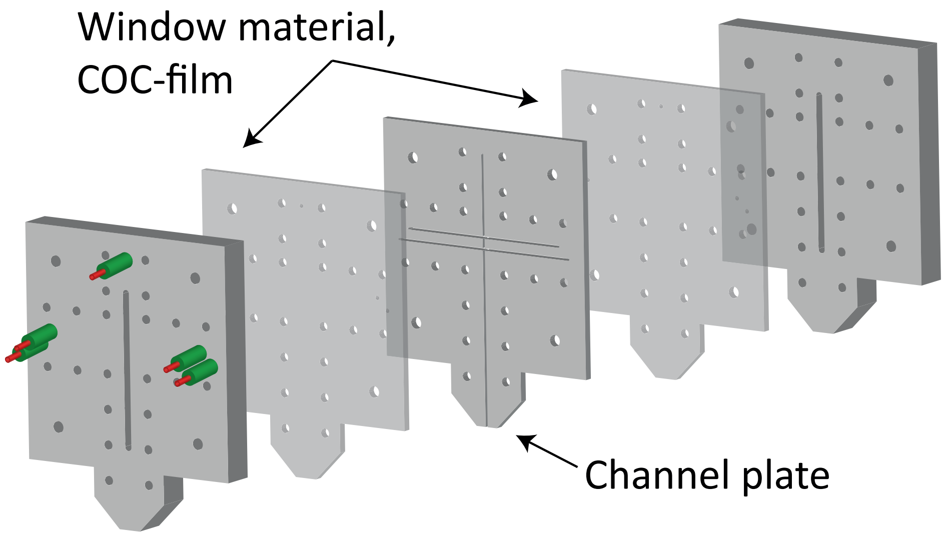

Figure 1: Illustration of the flow cell consisting of an aluminum channel plate with two transparent COC-films and two outer thicker aluminum plates that are all mounted together.

Flow cell

Fig 1 shows the flow cell used for the experiments. The double-focusing channel is milled out of a 1 mm thick aluminum plate at the center. It is sandiwiched between two COC-films that act as walls to the flow and provide optical access to the mixing region. The sandwich is mounted with two outer 10 mm thick aluminum plates where connections for the fluid are attached at the front plate facing the X-ray beam.

Flow setup

Fig 2 shows the setup of the flow equipment. The main flow (with flow rates Q_1, Q_2 and Q_3) is driven by three syringe pumps (NE-4000), where the sheath flow pumps have two 1 mL syringes each (one for each side channel) and the core flow naturally only needs one (1 mL) syringe. Two more syringe pumps holding larger 20 mL syringes are connected to the core and 2nd sheath flow respectively. These are primarily used to be able to push out small bubbles and fill up the tubing prior to the experiment and then kept turned off. For the 1st sheath flow with water, one syringe pump with two large 60 mL syringes are connected, each

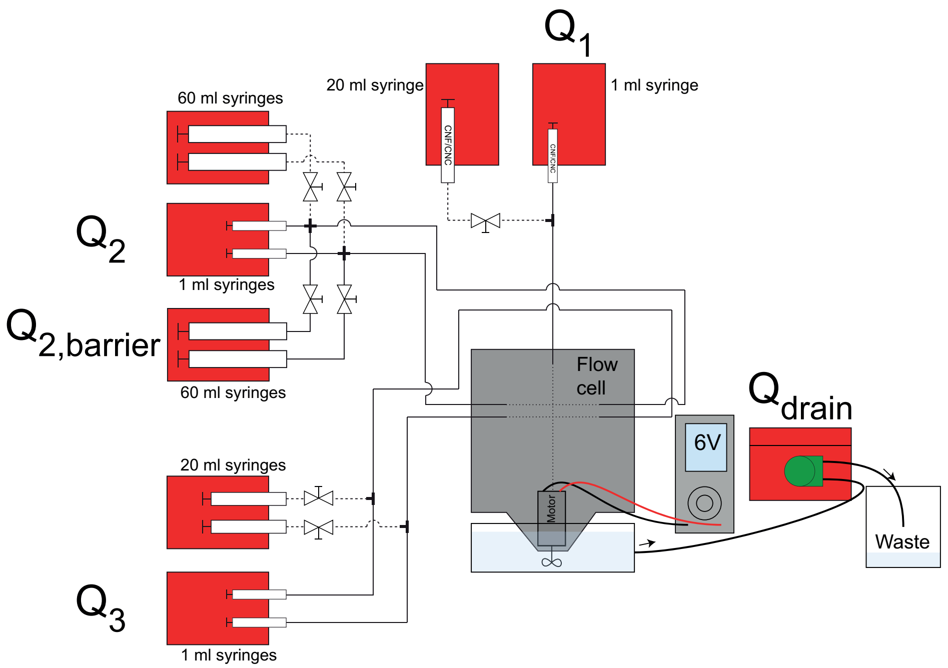

Figure 2: Illustration of the fluid distribution to the experiments. The main flow is driven by small 1 mL syringes to be able to run at low flow rates. Larger syringes are also connected to the system to be able to flush the flow cell with larger volumes and higher flow rates. A peristaltic pump is connected to maintain a constant level in the outlet container and a small motor is attached to the outlet to remove excess gel at the outlet. The pumps flow rates $Q_1, Q_2, Q_{2,\text{barrier}}, Q_3$ and Q_{drain} can be controlled remotely.

with flow rate $Q_{2,\text{barrier}}/2$. Their primary usage is to be able to run the flow continuously with a thick diffusion (barrier) layer between the salt solution and the CNC dispersion and thus avoid gelation in the channel while not measuring. They are also used to flush the channel with high flow rates to push out any accumulated gel inside the channel. For all larger pumps, manual shut-off valves are placed to increase the stiffness of the system and avoid potential bubbles in the large syringes to store energy and act as springs in the fluidic system if pressure would build up. Adding this was generally seen to lead to a more stable flow over a longer time period. During experiment all valves were shut, except for the valves to one of the 1st sheath flow pumps, in order to quickly flush the system.

Since the outlet container is constantly filled with the fluids from the flow cell, it needs to be drained using a peristaltic pump with flow rate $Q_{\text{drain}} = 5Q + Q_{2,\text{barrier}}$ to keep the level stationary. As gel is accumulating at the outlet, a small 6V motor rotating at around

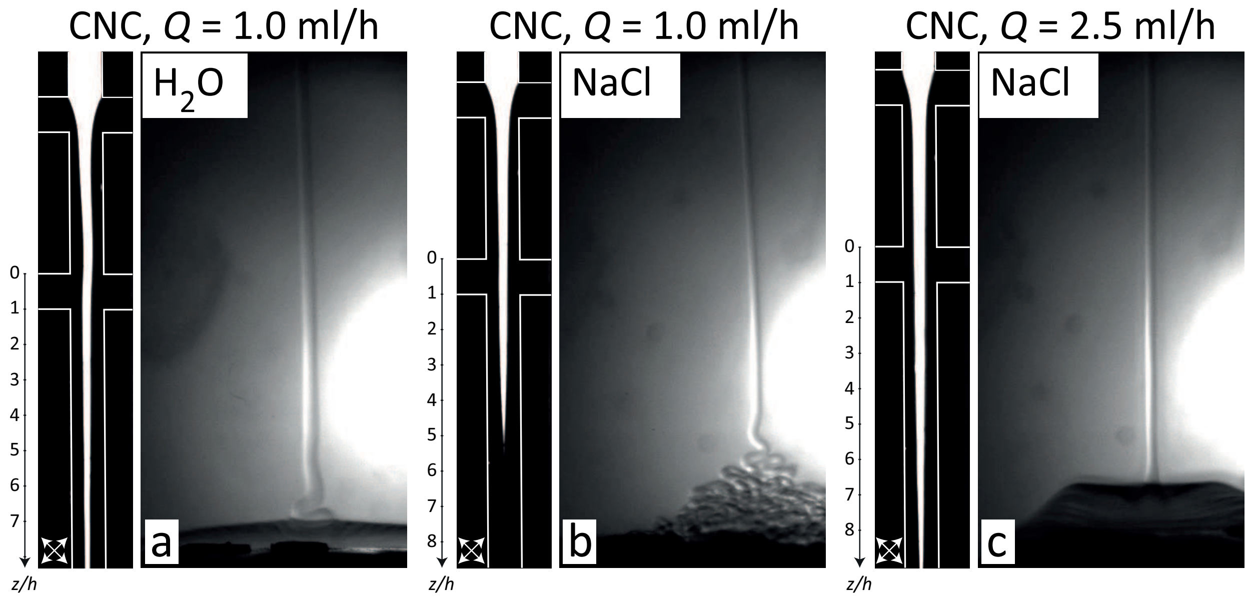

Figure 3: Images from the *in situ* polarized optical microscopy (POM) experiments (corresponding videos provided as Supplementary Information (SI)). Left side shows the POM intensity inside the channel and right side shows the CNC dispersion exiting the outlet. (a) $Q = 1$ mL/h with no gelation (water in the 2nd sheath flow). (b) $Q = 1$ mL/h with the NaCl solution as gelation agent. (c) $Q = 2.5$ mL/h with the NaCl solution as gelation agent.

5 rpm is attached to the flow cell to slowly remove gel without disturbing the flow upstream.

During the scanning-SAXS experiments, the three pumps with the 1 mL syringes (flow rates Q_1, Q_2 and Q_3) as well as the additional pump for the 1st sheath flow (flow rate $Q_{2,\mathrm{barrier}}$) and the peristaltic pump are controlled remotely from outside the experimental hutch.

In between each measurement, the flow rate of the 1st sheath is increased by setting $Q_{2,\mathrm{barrier}}/2 = 6.7$ mL/h, to ensure no gelation in the channel. When measuring, $Q_{2,\mathrm{barrier}}$ is set to zero and the flow is allowed to stabilize with the desired rates for the experiment. Directly after the measurement, to remove possible gel that might have accumulated, the channel is flushed with $Q_{2,\mathrm{barrier}}/2 = 5000$ mL/h for 1 s.

The system is running continuously with given flow rates Q_1, Q_2 and Q_3 ($Q_{2,\mathrm{barrier}} = 0$), while data is collected from the different y- and z-locations in the flow.

Supporting POM experiments

Prior to the SAXS experiment, the flow was studied with polarized optical microscopy (POM) to ensure that we indeed have a gel forming at the given flow conditions as well as determining the true core flow radius R_1 and velocity V. Fig. 3 shows images from the POM experiment with the left side showing the flow-induced birefringence (due to hydrodynamically aligned CNCs) in the channel and the right side showing the flow as it exists the flow cell into an outlet container with water. A supplementary video illustrating these experiments is provided in Supplementary Information.

By running only water in the second sheath flow and thus not inducing any gelation, we can observe that CNCs remain non-isotropic throughout the visible region (see Fig. 3a). As the CNC dispersion exits, we still observe the dispersion interface, but it is clearly not held together strongly and eventually diffuse out to the surrounding water. With gelation (*i.e.* with the NaCl solution in the 2nd sheath) in Fig. 3b, we can clearly observe an ion-induced loss of alignment at $z/h \approx 5$ and a gel thread exiting the channel and accumulating in the container. Running the system at a higher flow rate $Q = 2.5$ mL/h (instead of $Q = 1$ mL/h), we find that the point of isotropy in the channel is pushed downstream. Even though gel is accumulating in the container, the disappearance of the interface of the gel thread indicates that the gel is actually formed on the bottom of the container rather than inside the flow cell.

Owing to small impurities in the dispersions, it is straightforward to confirm that the flow is indeed a plug flow (all impurities moving with a constant velocity) as well as to experimentally determine the core velocity V and the projected radius $R_{1,y}$. By knowing the core flow rate Q_1, we can calculate the core radius in the viewing direction $R_{1,x}$ through $Q_1 = \pi R_{1,x} R_{1,y} V$. An illustration of the procedure is included in the supplementary video.

Estimating the ion concentration distribution in flow-focusing mixing

Predicting core radius and velocity

In order to predict the concentration distribution without an experimental measurement of the core radius $R_{1,y}$ or velocity V, these parameters can be estimated through the following procedure, which is also applied in the main manuscript for the prediction in Fig. 3.

The flow situation downstream of the focusing section in the double flow-focusing mixing cell can be estimated given three assumptions:

1. The flow with quadratic cross-section of $h \times h$, can be approximated with a cylindrical geometry with equivalent radius $R = h/\sqrt{\pi}$. This ensures the same average velocity for a given flow rate.

2. The flow consists of a core flow with flow rate Q_1 and radius R_1 surrounded by an inner sheath flow with flow rate Q_2 and radius R_2 and outer sheath flow with flow rate Q_3 bounded by the outer walls with the equivalent radius R.

3. The flow in the core is constant, i.e. $v(r \leq R_1) = V$. The flow outside of the core is described with a linear shear profile:

$$v(R_1 \leq r \leq R) = V \frac{r - R}{R_1 - R} \tag{1}$$

Given these assumptions, the unknowns R_1, R_2 and V can be calculated by knowing the flow rates Q_1, Q_2 and Q_3 in two steps.

Firstly, the radius R_1 can be calculated by knowing that:

$$Q_2 + Q_3 = 2\pi V \int_{R_1}^{R} \frac{r - R}{R_1 - R} r dr, \tag{2}$$

and that $Q_1 = V\pi R_1^2$, which leads to the expression:

$$Q_2 + Q_3 = \frac{Q_1}{R_1^2} \int_{R_1}^{R} \frac{r - R}{R_1 - R} r dr, \tag{3}$$

where R_1 is the only unknown and solution must fulfil $R_1 < R$. With R_1 known, the velocity V is found through $V = Q_1/(\pi R_1^2)$.

With these parameters known, the radius R_2 is found by using:

$$Q_2 = 2\pi V \int_{R_1}^{R_2} \frac{r - R}{R_1 - R} r dr, \tag{4}$$

with only R_2 unknown and finding the solution in interval $R_1 < R_2 < R$.

Simulations of the diffusion equation

Using the experimental values of V and R_1 (mean of $R_{1,x}$ and $R_{1,y}$), the time-scale analysis was performed (still keeping $R_2/h = 0.32$) as described in the main manuscript. This allowed for an estimation of the Na^+ concentration at different radial positions and downstream locations, which is plotted in Fig. 4a-b. From the simulated concentration profiles, three values were determined at different downstream positions: the centerline concentration (c_{CL}), the interface concentration at $r = R_1$ (c_{IF}) and the mean concentration in the core ($\langle c \rangle$). Compared to the simulation results in Fig. 3 in the main manuscript using the *predicted* values of core velocity V and radius R_1, the values are only slightly different. The mean Na^+ concentration distribution in Fig. 6 in the main manuscript is based on the *experimental* values of core velocity V and radius R_1.

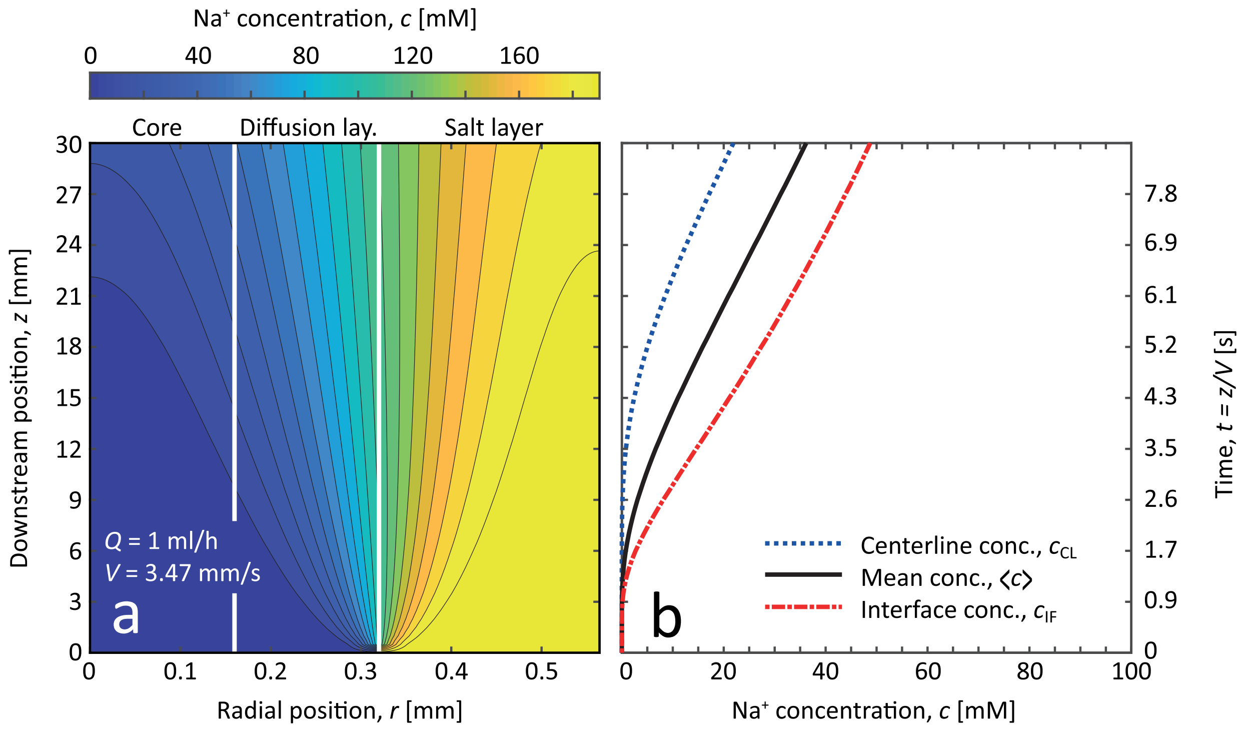

Figure 4: Concentration of Na^+ at different downstream locations z and radial positions r according to the procedure described in the main manuscript using the experimental values of core velocity V and radius R_1 ($R_2/h = 0.32$). (a) Concentration profile and (b) centerline, mean and interface concentrations at $Q = 3.47$ ml/h. Contours in (a) separated with 10 mM.

Experimental setup for SAXS experiments

Figure 5 shows the experimental setup of the *in situ* scanning-SAXS experiments at the LiX beamline, NSLS-II, Brookhaven National Laboratory, USA.

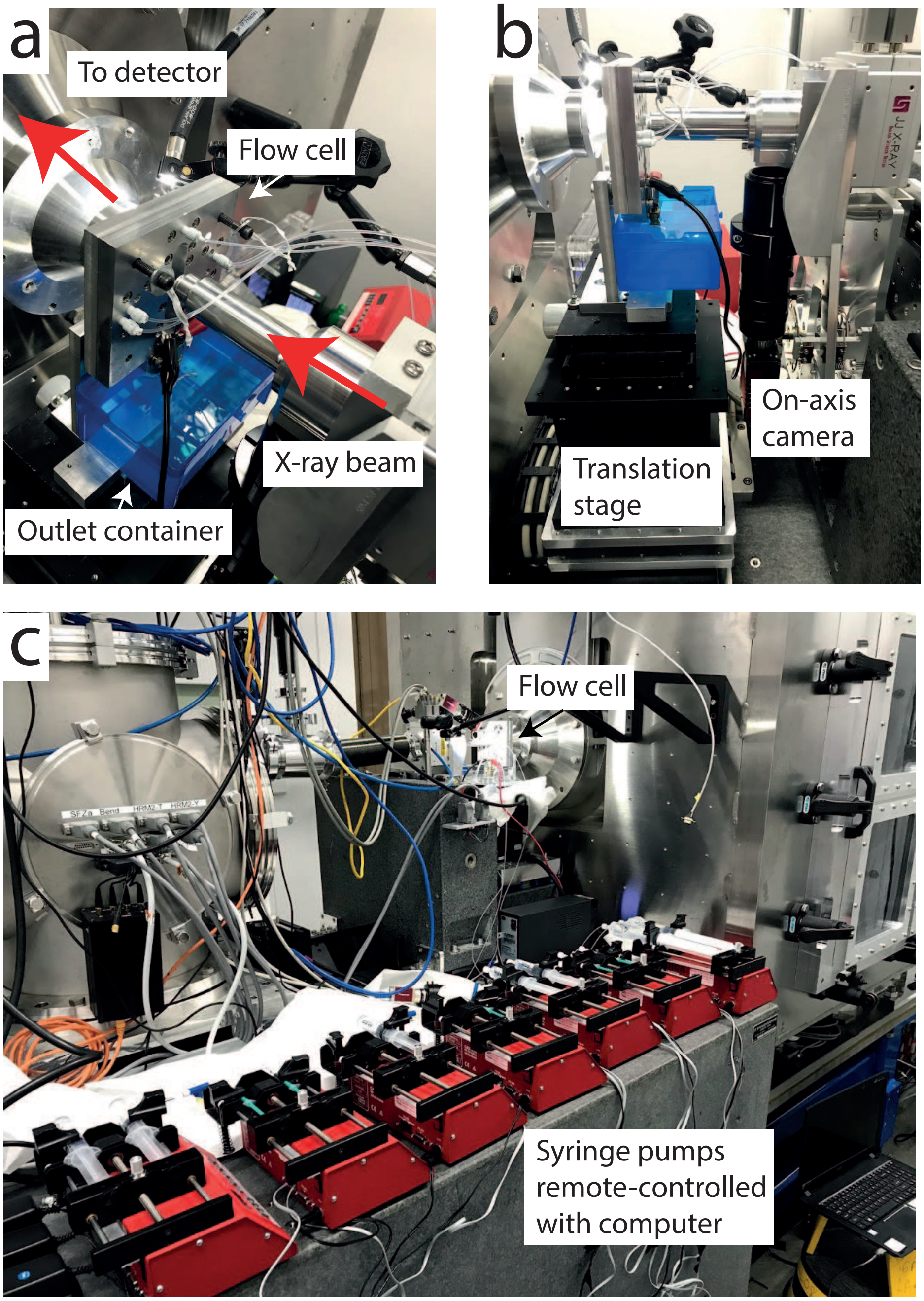

Figure 5: Photos from the experimental setup at the LiX beamline (16-ID), NSLS-II, Brookhaven National Laboratory, USA; (a)-(b) shows photos from different directions of the flow cell; (c) photo of the pump setup at the beamline.
